# Effect of intramuscular diazepam infusion on herpes zoster-related pain in older patients: a randomized, double-blind, placebo-controlled trial

**DOI:** 10.1186/s12871-024-02576-9

**Published:** 2024-05-29

**Authors:** Bingjie Ma, Meiling Xu, Lu Yang, Xuehua Huang, Peiliang Wang, Yun Ji, Ke Ma

**Affiliations:** 1 Department of Pain management, Xinhua Hospital Affiliated to Shanghai Jiaotong University School of Medicine, Shanghai, 200092 China; 2 Department of Pain management, The Fifth People’s Hospital of Qinghai Province, Xining city, 810007 Qinghai province China; 3 Department of Anesthesiology, Shanghai JiaoTong University Affiliated Sixth People’s Hospital, Shanghai, 200235 China

**Keywords:** Diazepam, Herpes zoster, Neuralgia, Sleep quality, Psychology

## Abstract

**Objectives:**

This study evaluated the effectiveness, psychological effects, and sleep quality using intramuscular diazepam infusion compared with placebo in patients with herpes zoster (HZ)-related pain.

**Methods:**

The patients were randomized to either the diazepam or control group. The diazepam group received an intramuscular injection of diazepam for 3 consecutive days, while the control group received an intramuscular injection of 0.9% normal saline. The primary outcome was pain relief on posttreatment day 4, as measured using the Visual Analog Scale (VAS). Moreover, anxiety and depression were evaluated using the Generalized Anxiety Disorder-7 (GAD7) and Patient Health Questionnaire-9 (PHQ9), respectively. Sleep quality was assessed using the Pittsburgh Sleep Quality Index (PSQI).

**Results:**

In total, 78 patients were enrolled in the trial. The mean differences in VAS scores between the two groups were 0.62 (*P* = 0.049) on posttreatment day 3 and 0.66 (*P* = 0.037) on posttreatment day 4. The effective rates of pain management in the diazepam group ranged from 10.26 to 66.67%, which were higher than those in the control group on posttreatment days 3 and 4 (*P* < 0.05). The mean difference in PSQI scores between the diazepam and control groups was 1.36 (*P* = 0.034) on posttreatment day 7. No differences were found in the incidence of analgesia-adverse 1reactions between the diazepam and placebo groups.

**Conclusions:**

The intramuscular injection of diazepam for 3 consecutive days provides effective pain management and improves the quality of life. Our study suggests that diazepam is more effective than the placebo in patients with HZ-related pain.

**Trial registration:**

The study was prospectively registered at https://www.isrctn.com/trialist(Registration date: 24/01/2018; Trial ID: ISRCTN12682696).

## Background

Herpes zoster (HZ) is caused by the reactivation of varicella zoster virus, which affects the sensory ganglia and their areas of innervation. HZ is characterized by severe neuralgic pain along the distribution of the affected nerve and crops of clustered vesicles over the area. A systematic review showed that the incidence rate of HZ ranged between 3/1000 and 5/1000 person-years. The incidence of HZ and the rate of HZ-associated complications increased with age, with 68% of cases occurring in those aged 50 years and older [1]. Postherpetic neuralgia (PHN) is the most common chronic complication of HZ and occurs in 20–50% of affected elderly patients [2, 3]. In China, the lifetime prevalence of HZ was 3.46% and the prevalence of PHN was 40% among people aged 50 and over [4]. Neurological symptoms include spontaneous pain, hyperalgesia, and allodynia, which seriously affect sleep, mood, and quality of life [5]. HZ-related pain is usually severe and hard to control. In order to reduce acute pain, paracetamol, NSAIDs, tramadol, opioids can be selected according to pain intensity [6]. In the management of PHN, tricyclic antidepressants, selective serotonin reuptake inhibitors, gabapentin, or pregabalin represent the treatment of first choice [7]. But these drugs usually have obvious side effects, including giddiness, constipation, nausea/vomiting, and so on. In addition, even with appropriate medicine, pain relief is usually unsatisfactory and severe pain is longstanding. Therefore, it is necessary to find new effective drugs to treat HZ-related pain.

Diazepam, a potent benzodiazepine, is a positive allosteric modulator (no functional response alone but increases the response of the endogenous ligand) of the γ-aminobutyric acid type A (GABA_A_) receptor complex that binds to a unique site on the alpha-gamma subunit interface. Diazepam binds to benzodiazepine receptors [8], which are voltage-gated sodium channels [9]. It also binds to a unique benzodiazepine receptor, initially named peripheral benzodiazepine receptor but later called translocator protein (TSPO, 18 kDa), which appears to be localized on the mitochondrial membrane [10]. Clinically, diazepam is used to treat anxiety, acute alcohol withdrawal, skeletal muscle spasm, convulsive disorders (e.g., status epilepticus), insomnia, restless leg syndrome, and pre/postoperative sedation. There were some trials that diazepam was applied in the domain of pain. Some studies indicated that oral diazepam could reduce pain during different invasive procedures [11, 12]. One study showed that oral diazepam was no better than the placebo when combined with naproxen for acute lower back pain [13]. Clonazepam (other benzodiazepines) could ameliorate pain intensity in patients with burning mouth syndrome [14]. The use of diazepam to treat HZ has rarely been reported worldwide. In clinical practice, we found that intramuscular diazepam could mitigate pain intensity and reduce the number of breakthrough pain in patients with HZ. Therefore, this study explored whether intramuscular diazepam would provide better analgesic and other positive effects. The primary outcome was pain scores on posttreatment day 4, as measured using the Visual Analog Scale (VAS).

## Materials and methods

### Patients

Patients who were diagnosed with HZ-related pain persisting beyond acute phase were enrolled between July 1, 2018, and January 31, 2020. This study was approved by the institutional ethics committee (Approval No: XHEC-C-2017-105-2) and prospectively registered at https://www.isrctn.com/trialist. The inclusion criteria were as follows: age ≥ 50 years, with HZ-related pain persisting for 30 to 180 days; a VAS (100-mm unmarked line, with anchors: 0 = no pain and 100 mm = worst pain imaginable) score ≥ 40 mm or the number of breakthrough pain ≥ 3/day. The exclusion criteria were as follows: severe heart, lung, liver, and kidney diseases, myasthenia gravis, sleep apnea syndrome, a history of benzodiazepine allergy and refusal to follow-up. Patients who suffered from HZ-related pain persisting beyond acute phase at Xinhua Hospital were randomly assigned to the diazepam or control group.

### Randomization and intervention

Participants were randomized by an investigator at a 1:1 ratio by generating sequences using SAS 9.3 software. The generated numbers were concealed in sequentially numbered envelopes. A doctor (Dr. Xu) drew the random numbers contained by envelopes and checked them on a random number table. Then, she allocated either diazepam or placebo treatment to each patient. The diazepam group received 5 mg/mL diazepam (2 mL, 10 mg), whereas a corresponding amount of saline (2 mL) was administered in the control group. A registered nurse (Ms. Hu) checked the basic information of the patients and administered the intramuscular infusion at 9:00 pm for three consecutive days. All patients were blinded to their group allocation.

During the study, all patients received the standard oral medicines (150 mg pregabalin 2 times daily; and 0.5 mg mecobalamin 3 times daily). The rescue analgesic (oxycodone-acetaminophen) was used when the VAS score ≥ 40 mm. Nerve block and other invasive interventional treatments were considered 4 weeks after the infusion if the patients reported no pain relief or worse pain.

#### Blinding

A doctor performed the randomization process and prepared all the solutions. All the other doctors, patients, medical staff members, and statistician were blinded to the treatment for each group.

#### Outcome evaluations

The primary outcome included pain scores on posttreatment day 4, as measured using VAS. The pain scores were obtained every 6 h and averaged for each 24-hour period posttreatment. The effective rate of pain was a percentage of pain relief greater than 50%. The secondary outcomes included pain-related outcomes, mental health, sleep quality, and safety. Pain-related outcomes included VAS scores measured at other time points, neuropathic pain measured using Douleur Neuropathique 4 questions (DN4), and rescue analgesics. Mental health outcomes included anxiety and depression at every time point during the first posttreatment month. Safety-related outcomes included the assessment of adverse effects. In the study, Generalized Anxiety Disorder-7 (GAD7) was used to evaluate anxiety, Patient Health Questionnaire-9 (PHQ9) was used to assess depression, and the Pittsburgh Sleep Quality Index (PSQI) was used to evaluate sleep quality.

### Postoperative follow-up

The VAS pain scores and DN4 scores were assessed on pretreatment day 1 (baseline) and posttreatment days 1, 2, 3, 4, 7, 14, and 28. The anxiety score, depression score, and quality of sleep were measured at baseline and on posttreatment days 7, 14, and 28. After discharge, the patients were followed up for 4 weeks at the outpatient clinic or by telephone or WeChat.

### Sample size calculation

PASS (version 15) was used to calculate the required sample size based on the pain scores obtained on posttreatment day 4 as the primary outcome variable. The results of the preliminary study showed that the difference in the pain score on posttreatment day 4 between the two groups was 0.7. The standard deviation (SD) for the diazepam group was 0.96, whereas it was 0.95 for the control group. The power of test 1-β was 0.8. Using the one-sided value of α = 0.025, the sample size of each group should be at least 31. Accordingly, the study enrolled 78 patients and obtained a dropout rate of 20%.

### Statistical analysis

Statistical analysis was performed using SPSS (version 22). The data collected included both continuous and categorical variables and were evaluated for normality using the Kolmogorov–Smirnov test. Normal quantitative data were expressed as mean and SD, whereas nonnormal quantitative data were expressed as median and IQR. Repeated-measures ANOVA was conducted to compare the changes in VAS pain scores between baseline and those on posttreatment days 1, 2, 3, 4, 7, 14, and 28. The same statistical analyses were used to evaluate anxiety, depression, and quality of sleep scores. The Mann–Whitney U-test was used to analyze rescue analgesic use. Age, body mass index and course of disease were compared using two-tailed t-tests, whereas the categorical variables were compared using the two-tailed Fisher’s exact test. *P* < 0.05 was considered statistically significant. Data are expressed as ratio (percentage), mean (SD), or median (IQR).

## Results

**Fig. 1:**
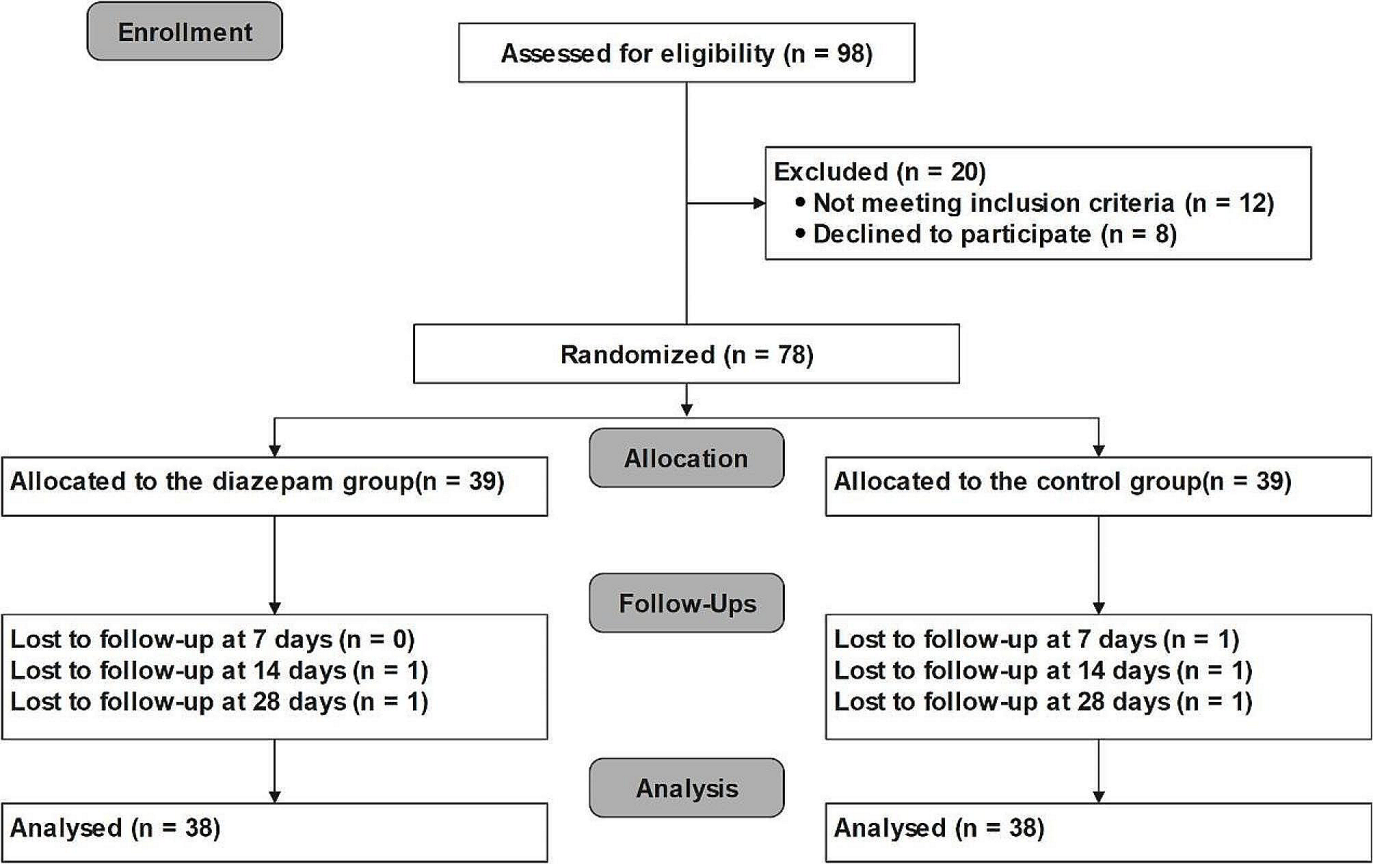
Participant flow diagram of the study

Of the 98 enrolled patients, 20 were excluded. Eventually, 78 patients were finally randomized to either the diazepam (*n* = 39) or control group (*n* = 39; Fig. 1). No differences were found in the baseline demographic characteristics between the two groups (Table 1). In the study, only 5 patients experienced breakthrough pain; thus, thereby removing the need to perform the relevant statistical analysis.

**Table 1:** Demographic and baseline characteristics

	Control group	Diazepam group	*P*
Age, years	68.38 ± 9.38	66.08 ± 8.91	0.813
Female sex, n (%)	20(51.28%)	19(48.72%)	0.821
BMI, kg/m2	24.49 ± 3.59	23.24 ± 2.58	0.315
**Comorbidities**, n (%)			
Hypertension	9(23.08%)	7(17.95%)	0.575
Diabetes mellitus	2(5.13%)	3(7.69%)	1
Coronary artery disease	0	3(7.69%)	0.239
Tumor/autoimmune disease	1(2.56%)	2(5.13%)	0.792
Location of rash, n (%)			
Craniofacial	5(12.82%)	4(10.26%)	1
Cervical	7(17.95%)	9(23.08%)	0.575
Thoracic	23(58.97%)	22(56.41%)	0.819
Lumbar	3(7.69%)	3(7.69%)	1
Sacral	1(2.56%)	1(2.56%)	1
Course of disease, day	44.03 ± 17.84	46.59 ± 18.44	0.749
A history of minimally invasive treatment for herpes zoster neuralgia	8(20.5%)	10(25.6%)	0.591

**Fig. 2:**
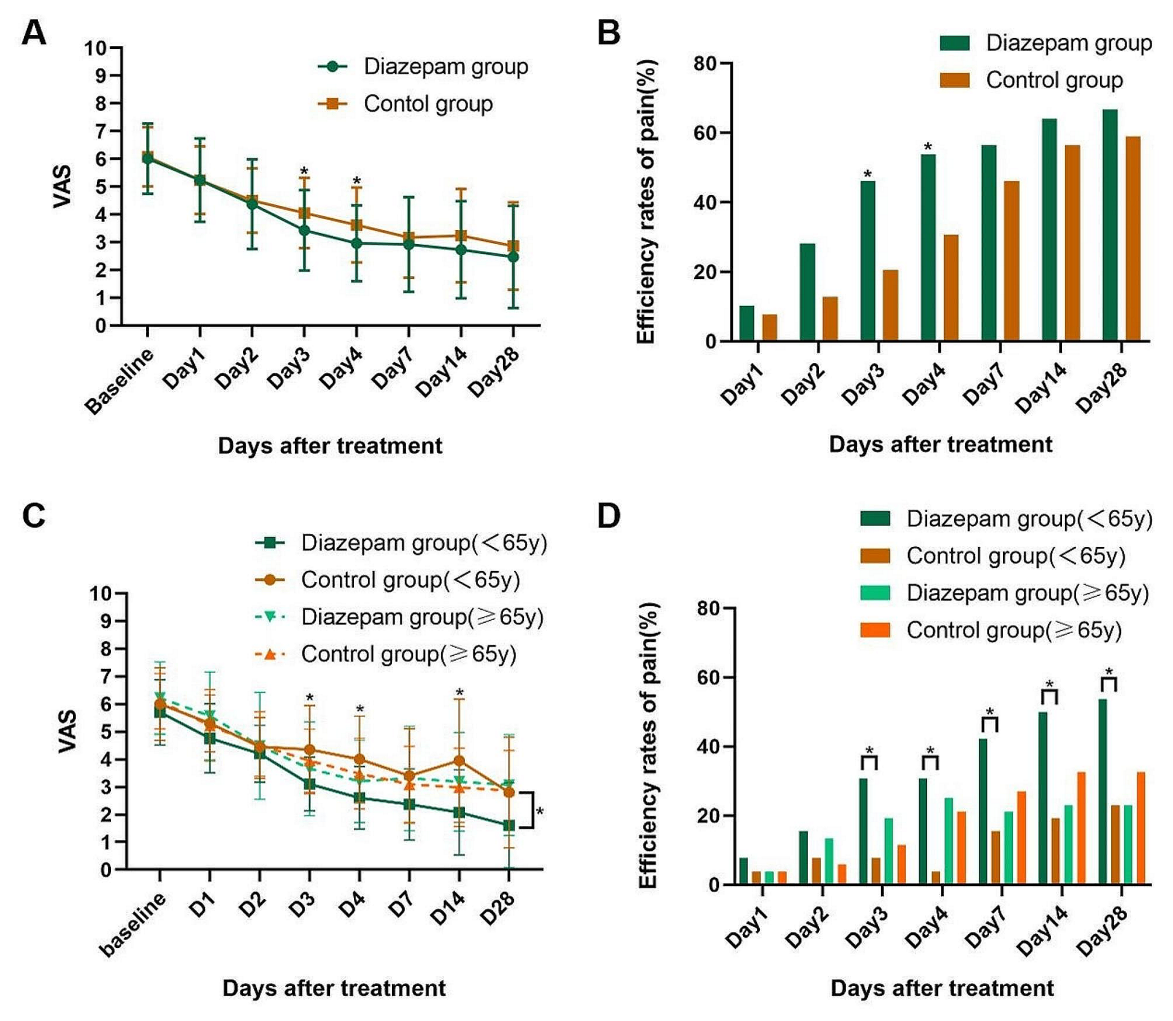
(**A-D**). Pain conditions in patients with HZ-related pain between the diazepam and control group. (**A**) VAS scores at different time points; (**B**) Efficiency rates of pain (VAS scores decreased ≥ 50%) at different time points; (**C**) VAS scores at different time points(age<65 years, age ≥ 65 years); (**D**) Efficiency rates of pain (VAS scores decreased ≥ 50%) at different time points(age<65 years, age ≥ 65 years). Bars represent SD; ∗∗*P* < 0.01 and ∗*P* < 0.05. VAS: Visual Analog Score

The pretreatment VAS scores of the two groups were 6.01 and 6.08, respectively. The mean difference in VAS score between the two groups was 0.62 (*P* = 0.049) on posttreatment day 3 and 0.66 (*P* = 0.037) on posttreatment day 4 (Fig. 2A). The VAS scores at the other time points were similar between the two groups. The pain scores after treatment were significantly lower than the baseline in both groups (*P* < 0.01). A significantly greater proportion of patients who received diazepam therapy reported ≥ 50% pain relief compared with the control group on posttreatment days 3 and 4 (Day 3: 18/39 vs. 8/39, *P* = 0.016; Day 4: 21/39 vs. 12/39, *P* = 0.039; Fig. 2B). To delve deeper into the varying effects across different age cohorts, we segregated patients in each group into two subgroups using a cutoff age of 65. Our analysis revealed no significant differences between the two groups (age ≥ 65 years) across all time points. However, there were statistically significant differences observed in the main effect ‘group’ between the two groups (age < 65 years) (*P* = 0.02). The mean difference in VAS score between the two groups(age<65 years ) was 1.24 (*P* = 0.021) on posttreatment day 3, 1.39 (*P* = 0.014) on posttreatment day 4 and 1.97 (*P* = 0.018) on posttreatment day 14 (Fig. 2C). Additionally, a notably higher proportion of patients receiving diazepam therapy reported ≥ 50% pain relief compared to the control group (age < 65 years) on posttreatment days 3, 4, 7, 14, and 28 (Day 3: 8/26 vs. 2/26, *P* = 0.035; Day 4: 8/26 vs. 1/26, *P* = 0.028; Day 7: 11/26 vs. 4/26, *P* = 0.032; Day 14: 13/26 vs. 5/26, *P* = 0.02; Day 28: 14/26 vs. 6/26, *P* = 0.023; Fig. 2D).

**Fig. 3:**
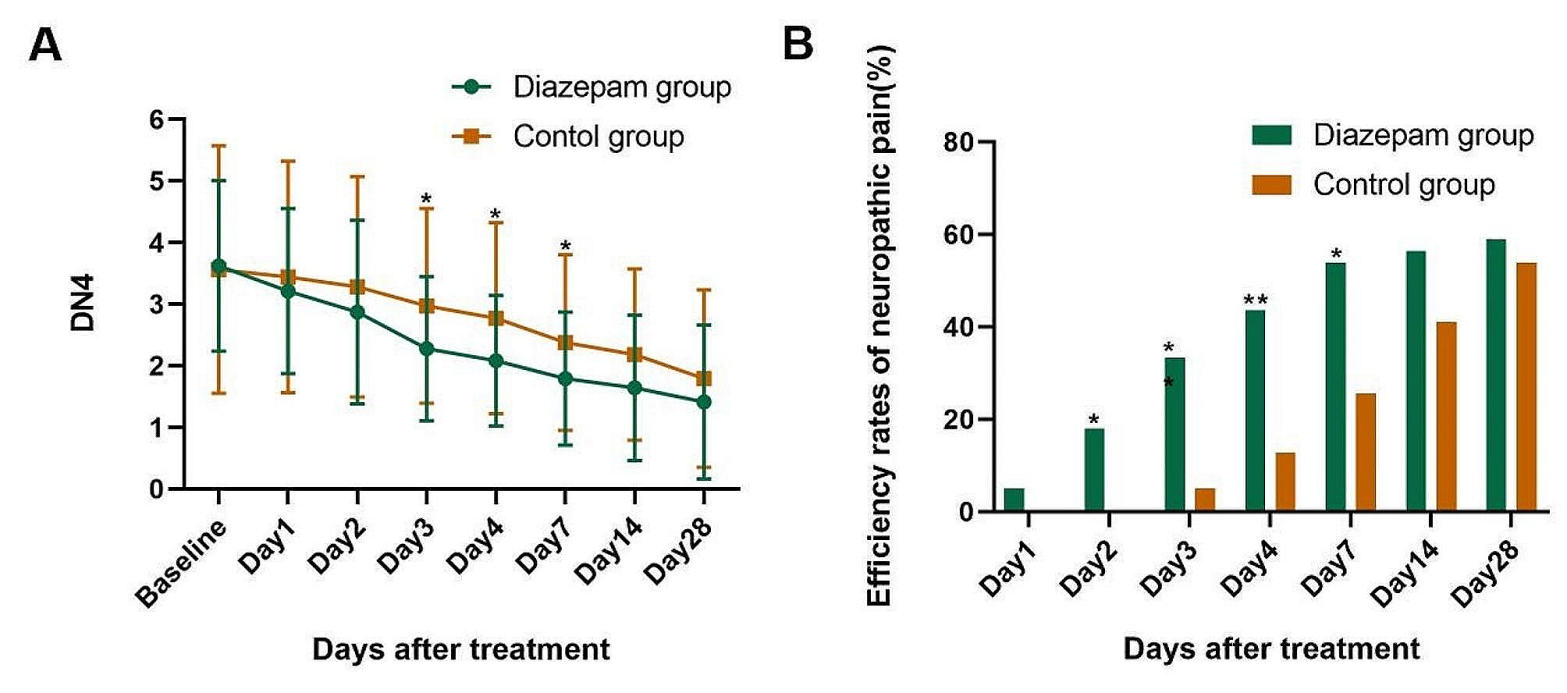
(**A-B**). Neuropathic pain conditions in patients with HZ-related pain between the diazepam and control group. (**A**) Neuropathic pain conditions (DN4 scores) at different time points; (**B**) Efficiency rates of neuropathic pain (DN4 scores decreased ≥ 50%) at different time points. Bars represent SD; ∗∗*P* < 0.01 and ∗*P* < 0.05. DN4: Douleur Neuropathique 4 questions

The mean difference in DN4 scores between the two groups was 0.69 (*P* = 0.031) on posttreatment day 3, 0.69 (*P* = 0.024) on posttreatment day 4, and 0.59 (*P* = 0.043) on posttreatment day 7 (Fig. 3A). The DN4 scores on all the other days were similar. We further assessed the degree of improvement in the DN4 scores and found that more patients in the diazepam group had a decrease of ≥ 50% in DN4 scores compared with the control group on posttreatment days 2, 3, 4, and 7 (Day 2: 0/39 vs. 7/39, *P* = 0.017; Day 3: 2/39 vs.13/39, *P* = 0.004; Day 4: 5/39 vs. 17/39, *P* = 0.003; Day 7: 10/39 vs. 21/39, *P* = 0.011; Fig. 3B).

**Fig. 4:**
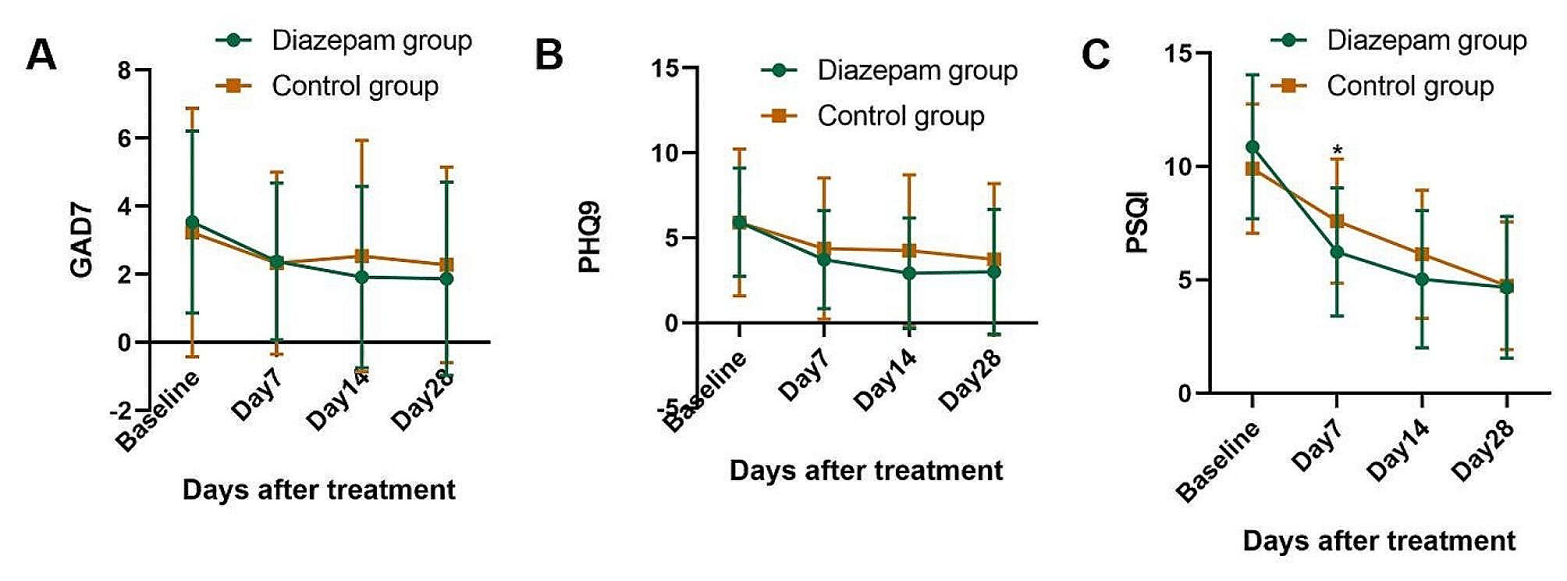
(**A-C**). (**A**)Anxiety scores (GAD7), (**B**)depression scores (PHQ9), and (**C**)sleep quality scores (PSQI) in patients with HZ-related pain between the diazepam and control group. Bars represent SD; ∗∗*P* < 0.01 and ∗*P* < 0.05. GAD7, Generalized Anxiety Disorder-7SCARED; PHQ9, Patient Health Questionnaire-9; PSQI, Sleep quality was assessed using the Pittsburgh Sleep Quality Index

The GAD7 scores improved from posttreatment day 14 in the diazepam group, whereas they were similar at all-time points in the control group. However, no significant differences in the GAD7 scores were observed between the 2 groups (Fig. 4A).

Mild depression was assessed in both groups before treatment through the PHQ9 (5.90 vs. 5.92). Both groups showed improved PHQ9 scores from posttreatment day 7 (*P* < 0.05), but no significant differences were observed between the two groups at all-time points (Fig. 4B).

The mean difference in PSQI scores between the diazepam and control groups was 1.36 (*P* = 0.034) on posttreatment day 7 (Fig. 4C). Otherwise, the PSQI scores were similar on all other days. The PSQI scores of the two groups improved from posttreatment day 7.

In the diazepam group, rescue analgesic use was lower on posttreatment days 4 and day 14 (Day 4: *P* = 0.047; Day 14: *P* = 0.042). On posttreatment day 14, more patients reduced their pregabalin use in the diazepam group (14/39 vs. 6/39, *P* = 0.038), whereas in the control group, more patients increased their use (2/39 vs. 9/39, *P* = 0.023). On posttreatment day 28, more patients reported decreased pregabalin use in the diazepam group (18/39 vs. 7/39, *P* = 0.008; Table 2). No significant difference was observed in the incidence of analgesia-adverse reactions between the diazepam and placebo groups at 28.21% and 23.08% respectively. Only a few patients reported nausea, vomiting, dizziness, or somnolence, and no serious adverse reactions were noted (Table 3).

**Table 2:** Change in analgesic use after diazepam infusion

	Diazepam group	Control group	*P*
rescue analgesics during hospital (pills)			
Post treatment day1	1.64 ± 1.11	1.85 ± 0.90	0.374
Post treatment day2	1.08 ± 1.11	1.23 ± 0.90	0.504
Post treatment day3	0.54 ± 0.79	0.82 ± 0.79	0.119
Post treatment day4	0.36 ± 0.74	0.72 ± 0.83	0.047
total	3.62 ± 3.04	4.64 ± 2.84	0.128
Analgesic Use after discharge(n)			
Increased pregabalin Post treatment day14	2/39	9/39	0.023
Decreased pregabalin Post treatment day14	14/39	6/39	0.038
Use of rescue analgesics Post treatment day14	2/39	8/39	0.042
Increased pregabalin Post treatment day28	2/39	6/39	0.263
Decreased pregabalin Post treatment day28	18/39	7/39	0.008
Use of rescue analgesics Post treatment day28	4/39	5/39	0.355

**Table 3:** Comparison of treatment related adverse events

	Diazepam group	Control group	*P* value
Total	11	9	0.604
Nausea, vomiting	2	2	1
dizziness	6	5	0.745
somnolence	3	2	1
confusion	0	0	/
sedation	0	0	/
respiratory depression	0	0	/
tachycardia	0	0	/

## Discussion

This prospective, randomized controlled study showed that the intramuscular injection of diazepam for 3 consecutive days alleviated VAS scores, DN4 scores, and oral analgesic use and improved the quality of life of patients with neuropathic pain.

In the current study, the diazepam group showed superior analgesic effects and, notably, an opioid-sparing effect. Furthermore, the VAS scores were lower on posttreatment days 3 and 4, while the DN4 scores were lower on posttreatment days 3, 4, and 7. No clinical trials have evaluated diazepam for the treatment of HZ-related pain. A study on vaginal diazepam and transcutaneous electrical nerve stimulation showed their efficacy in improving pain and alleviating vestibular hypersensitivity in women with vestibulodynia [15]. A study showed that intrathecal midazolam, which is also a benzodiazepine, provided short-term improvement in pain and allodynia in patients with PHN [16]. We suspect that the pharmacological properties of midazolam and diazepam, and thus their analgesic effects, are similar. One study demonstrated that intraperitoneal administration of diazepam in rats markedly attenuated mechanical allodynia and thermal hyperalgesia [17]. Additionally, another study indicated that spinal diazepam administration partially mitigated neuropathic hypersensitivity, including spontaneous pain, allodynia, and hyperalgesia [18]. Thermal hyperalgesia, allodynia and hyperalgesia are integral components of DN4 scores. Remimazolam, a benzodiazepine derivative, exerts analgesic effects on neuropathic pain by modulating the bradykinin receptor B1 and influencing autophagy pathways [19]. Diazepam could modulate the GABA receptor to enhance GABA activity and inhibit pain transmission pathways in the spinal cord. The administration of GABA antagonists stimulates severe pain, whereas that of GABA neurotransmitters promotes pain relief [20]. Diazepam also binds to the unique benzodiazepine receptor TSPO, whose agonist could alleviate neuropathic pain [21, 22]. An animal trial showed that a single intraperitoneal injection of diazepam could inhibit neuropathic pain by binding TSPO [17].

HZ-related pain severely impacted patients’ sleep quality and mental health [23]. Sleep quality was improved in both groups with a statistical difference between the groups on posttreatment day 7. Additionally, diazepam exerts a hypnotic effect that can improve sleep. However, this may merely result from improvement in pain. The bidirectionality of the relationship between sleep and pain is widely accepted [24]. After treatment, the anxiety and depression scores improved in both groups, although no significant difference was found. As is well known, there’s a complex and bidirectional relationship between pain, anxiety and depression [25–28]. Chronic pain often co-occurs with anxiety and depression [29]. This complex relationship between pain and mental health disorders is due in part to the overlapping neural mechanisms by the observation from functional imaging studies [29–31]. Patients with postherpetic neuralgia had higher scores for anxiety and depression [32]. Although there’s no significant difference in anxiety and depression between the two groups in our study, but diazepam, as an anti-anxiety medication, reduced pain in the patients suffered from HZ-related pain. This maybe because of the complex relationship between anxiety and pain.

Acetaminophen, tramadol, and other opioids are usually used for HZ-related pain; however, they do not provide an adequate analgesic effect [6]. For patients with PHN, tricyclic antidepressants and anticonvulsants, such as amitriptyline and pregabalin, are the first-line medications [33, 34]. Despite their effectiveness, their adverse effects, including dizziness and somnolence, limit their use [35]. In our study, we conducted an analysis of analgesic consumption within the allowed dosage range. Patients in the diazepam group required lower amounts of oxycodone-acetaminophen at 4 days posttreatment. In addition, more patients in the diazepam group reduced the dose or ceased taking analgesics, whereas patients in the control group required higher amounts of pregabalin and oxycodone-acetaminophen at 2–4 weeks posttreatment. No significant difference in the incidence of analgesia-associated adverse reactions was observed between the two groups. In the diazepam group, only a few patients reported nausea, vomiting, dizziness, and hypersomnia, indicating the safety of intramuscular diazepam.

The study has some limitations. First, all 78 patients in the study were enrolled from the same center. Thus, a future multicenter study with a larger sample size needs to be conducted. Second, rescue analgesics were administered, which could be a confounding factor in analyzing the efficacy and safety of diazepam. Third, our primary outcome was the pain scores on posttreatment day 4, which were used to calculate the required sample size. Our study thus probably lacked sufficient statistical power (not enough patients included) to evaluate differences in other items.

## Conclusions

The intramuscular injection of diazepam for 3 consecutive days alleviated pain and improved the quality of life. Our study suggests that diazepam is more effective than the placebo in patients with HZ-related pain.

## Data Availability

The original data and materials used to support the findings of this study are available from the corresponding author upon request.

## Abbreviations

HZ: herpes zoster
PHN: Postherpetic neuralgia
GABA_A_: γ-aminobutyric acid type A
TSPO: translocator protein
VAS: Visual Analog Scale
DN4: Neuropathique 4 questions
GAD7: Generalized Anxiety Disorder-7
PHQ9: Patient Health Questionnaire-9
PSQI: Pittsburgh Sleep Quality Index
